# Association between the exposure to anti-angiogenic agents and tumour immune microenvironment in advanced gastrointestinal stromal tumours

**DOI:** 10.1038/s41416-019-0596-1

**Published:** 2019-10-14

**Authors:** Changhoon Yoo, Yeon-mi Ryu, Sang-Yeob Kim, Jihun Kim, Chan Young Ock, Min-Hee Ryu, Yoon-Koo Kang

**Affiliations:** 1 Department of Oncology, Asan Medical Center, University of Ulsan College of Medicine, Seoul, Korea; 2 Asan Institute for Life Sciences, Asan Medical Center, University of Ulsan College of Medicine, Seoul, Korea; 3 Department of Convergence Medicine, Asan Medical Center, University of Ulsan College of Medicine, Seoul, Korea; 4 Department of Pathology, Asan Medical Center, University of Ulsan College of Medicine, Seoul, Korea; 5 Theragen Etex Bio Institute, Suwon, Korea

**Keywords:** Sarcoma, Cancer microenvironment

## Abstract

**Background:**

Tumour immune microenvironment (TIME) of gastrointestinal stromal tumours (GISTs) is largely unknown.

**Methods:**

A total of 81 surgical specimens from 67 patients with advanced GISTs were categorised into treatment groups: tyrosine kinase inhibitor (TKI)-naive, *n* = 20; imatinib-progressed and no exposure to sunitinib or regorafenib (IM-PD), *n* = 30; and imatinib-progressed and sunitinib and/or regorafenib-treated (IM-PD/SU-treated), *n* = 31. Multiplexed immunofluorescence staining and RNA sequencing were performed to define TIME.

**Results:**

PD-L1 expression rate (>1%) of DOG-1^+^ tumour cells was 5.0, 6.7, and 29.0% in TKI-naive, IM-PD, and IM-PD/SU-treated group, respectively (*p* = 0.02). FoxP3 expression of CD3^+^ T cells and CD204^+^ CD68^+^ monocytes per DOG-1^+^ cells was significantly higher in IM-PD/SU-treated group compared to TKI-naive and IM-PD groups (*p* < 0.05). IM-PD/SU-treated group showed increased expression of PD-1 on CD3^+^ T cells (*p* = 0.03 vs TKI-naive; p = 0.003 vs IM-PD) and DOG-1^+^ tumour cells (*p* = 0^.^02 vs TKI-naive; *p* = 0.006 vs IM-PD), TIM-3 expression on CD3^+^ T cells (*p* = 0.01 vs TKI-naive; *p* = 0.002 vs IM-PD), and LAG3 expression on CD3^+^ T cells (*p* = 0.001 vs TKI-naive; *p* = 0.004 vs IM-PD). In the RNAseq analysis, *TIGIT* expression was significantly increased in IM-PD/SU-treated GISTs compared to IM-PD (*p* = 0.01).

**Conclusion:**

Immunosuppressive phenotype was predominant in tumours treated with anti-angiogenic agents compared to TKI-naive and IM-treated tumours.

## Background

Gastrointestinal stromal tumours (GISTs) are the most common mesenchymal tumours of the digestive tract and commonly occur in the stomach and small intestine.^1^ The molecular characteristics of GISTs include *KIT* or *platelet-derived growth factor receptor alpha* (*PDGFRA*) mutations as single driver mutations, which are detectable in >90% of cases.^1^ These unique characteristics are associated with the dramatic improvement of clinical outcomes after the introduction of tyrosine kinase inhibitor (TKI).^1^

Imatinib (IM) is an oral TKI with activity against KIT, PDGFRA, ABL, and DDR. The efficacy of imatinib was first demonstrated in the pivotal B2222 trial,^2^ which showed that the median time-to-progression (TTP) with imatinib was two years in the extended follow-up report.^3^ Sunitinib (SU) is the approved second-line therapy, with a median TTP of approximately seven months, as determined in the phase III trial.^4^ Regorafenib (REG) is the only approved drug as standard third-line therapy based on the success of a randomized phase III trial (GRID) that showed a median progression-free survival (PFS) of approximately 5 months in the regorafenib arm.^5^ For patients with refractory disease, re-challenge with IM demonstrated a significant delay of tumour progression compared to placebo in the RIGHT trial.^6^

Resistance to treatment with IM can be divided into primary and secondary resistance.^7^ Primary resistance defined as progression within first 6 months of treatment and the probability of primary resistance depends on the mutation profiles of tumour, as PDGFRA D842V mutation is strongly resistant to IM.^8^ Secondary resistance is the tumour progression after an initial benefit from IM and acquired secondary mutations are attributable most to this.^7^ Acquired secondary mutations commonly developed in KIT exon 13, 14, 17 and 18 showed different in vitro sensitivity for salvage TKI such as SU and REG, and this might relate with the diverse clinical outcomes in the salvage setting after progression on IM.^7^

Despite of recent promising data with novel agents such as avapritinib, ripretinib and cabozantinib on IM-refractory GISTs,^9,10^ current therapeutic strategies are mainly based on KIT inhibition. There are unmet clinical needs for different approaches to improve the survival outcomes of patients with advanced GISTs. Since the anti-CTLA-4 inhibitor ipilimumab was approved for the management of advanced melanoma,^9^ immune checkpoint inhibitors (ICIs), such as anti-cytotoxic T lymphocyte associated antigen 4 (CTLA-4) and anti-programmed cell death protein-1 (PD-1)/programmed death-ligand-1 (PD-L1) antibodies, have led a paradigm shift in the management of advanced cancers. They have shown remarkable improvement in survival outcomes, particularly in long-term survival.^10,11^ The United States (US) Food and Drug Administration (FDA) has approved ICIs for the management of various cancer types including melanoma, lung cancer, and hepatocellular carcinoma.

There is a lack of well-designed immunotherapy clinical trials for advanced GISTs, and no immunotherapeutic agent has been approved for the management of GISTs. Considering that many immunotherapeutic strategies are now under development, the evaluation of tumour immune microenvironment (TIME) of advanced GISTs is essential for the successful incorporation of immunotherapy in the management of GISTs. Although few prior studies showed the PD-L1 expression on tumour or immune cell in some GISTs,^12,13^ more extensive investigation using the diverse markers of TIME is needed for better understanding to make the strategies for future immunotherapy clinical trials.^14^ Furthermore, potential changes of TIME during the course of currently approved therapy are also important to estimate the optimal timing of the application of immunotherapy. Here, we evaluated key elements of TIME including tumour or immune cell expression of immune checkpoints, regulatory T cells and macrophages in advanced GISTs of different clinical settings.

## Methods

### Patients

Between May 2016 and October 2017, a total of 563 GISTs patients were enrolled in the TIME research program in Asan Medical Center, Seoul, Korea. Among them, 67 patients (81 surgical specimens) who had archival tissue specimens from surgery with appropriate quality for analysis were included in the current analysis, if specimens were acquired at the clinical setting of our research interest. Specimens were categorized in three groups: TKI-naive group (*n* = 20); IM-PD group for IM-progressed and no exposure to SU or REG (*n* = 30); and IM-PD/SU-treated group for IM-progressed and SU and/or REG treated (*n* = 31). Multiplexed immunofluorescence staining was performed for all 81 specimens, and RNAseq was done for 29 specimens (10 TKI-naive, 14 IM-PD, and five IM-PD/SU-treated group).

The study was approved by the Institutional Review Board of Asan Medical Center, Seoul, Korea and conducted in accordance with the Declaration of Helsinki and Good Clinical Practice. Informed consent for immunohistochemistry analysis was obtained before enrolment in this study.

### Multiplexed immunofluorescence staining

Four-micrometre-thick whole-slide sections, obtained with a microtome, were transferred onto positively-charged slides, followed by multiplexed immunofluorescence staining (Leica Bond Rx^TM^ Automated Stainer; Leica Biosystems, Newcastle, UK). Briefly, the slides were baked for 30 min and dewaxed (Leica Bond Dewax solution Cat #AR9222; Leica Biosystems, Milton Keynes, UK), followed by antigen retrieval (Bond Epitope Retrieval 2 Cat #AR9640; Leica Biosystems, Milton Keynes, UK) in a pH 9.0 solution for 30 min.

**Fig. 1:**
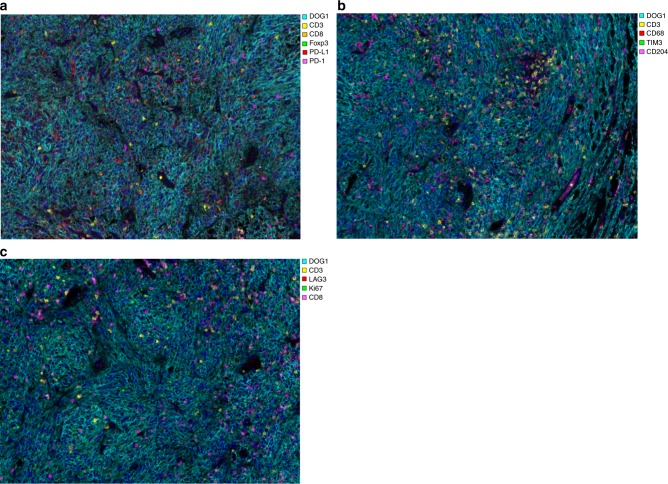
Representative examples of multiplexed staining of GISTs. **a** DOG-1, CD3, CD8, FoxP3, PD-L1, PD-1; **b** DOG-1, CD3, CD68, CD204, TIM-3; **c** DOG-1, CD3, CD8, LAG-3, Ki-67. ×200 magnification for all. GISTs, gastrointestinal stromal tumours

Three panels were designed for multiplexed immunofluorescence staining (Opal™ 7-color Automation IHC Kit; Perkin Elmer, Waltham, MA, USA): panel 1 (DAPI, DOG-1, CD3, CD8, FoxP3, PD-1, PD-L1), panel 2 (DAPI, DOG-1, CD3, T cell immunoglobulin-3 [TIM-3], CD68, CD204), and panel 3 (DOG-1, CD3, CD8, lymphocyte activation gene-3 [LAG-3], Ki-67) (Fig. 1). Further details of the panel information are summarised in Supplementary Table 1. DOG-1^+^ was used to indicate the tumour cells of GISTs; FoxP3^+^ CD3^+^ for regulatory T cells; CD204^+^ CD68^+^ for M2-polarised macrophages; and CD204^-^ CD68^+^ for M1-polarzied macrophages.

Each section was subjected to five or six sequential rounds of staining, each including a protein block with PKI blocking/antibody diluent, followed by incubation with primary antibody and corresponding secondary horseradish peroxidase-conjugated polymer (Opal^TM^ Polymer HRP Ms plus Rb kit; Perkin Elmer, Waltham, MA, USA). Each horseradish peroxidase-conjugated polymer mediated the covalent binding of a different fluorophore using tyramide signal amplification (TSA). This covalent reaction was followed by additional antigen retrieval (Bond Epitope Retrieval 1 Cat #AR9961; Leica Biosystems, Milton Keynes, UK) for 20 min to remove bound antibodies before the next step in the sequence. After sequential reactions, sections were counterstained with DAPI and coverslipped (HIGHDEF® IHC fluoromount; Enzo Life Sciences, Farmingdale, NY, USA).

### Multispectral imaging and analysis

Multiplex stained slides were acquired using Vectra® Polaris Quantitative Pathology Imaging System (Perkin Elmer, Boston, MA, USA). Each ×200 multispectral image cube was created by combining images obtained at 10-nm intervals of the emission light spectrum across the range of each emission filter cube. Filter cubes used for multispectral imaging were DAPI (440–680 nm), FITC (520–680 nm), Cy3 (570–690 nm), Texas Red (580–700 nm), and Cy5 (670–720 nm). In each slide, eight to 11 region of interests (ROIs) were selected, and images were spectrally unmixed and segmented (inForm 2.4.1 image analysis software; Perkin Elmer, Wellesley, MA, USA). Data obtained from inForm were sent to Spotfire™ software (TIBCO Software Inc., Palo Alto, CA, USA). Threshold for the positivity of each marker is determined by the pathologist using immunohistochemistry scoring; >0.2 for DOG, >3.0 for CD3, >1.5 for CD8, >1.2 for FoxP3, >1.7 for PD-L1, >0.5 for PD-1, >1.1 for LAG3, >5.0 for Ki-67, >0.7 for CD68, >0.5 for TIM3, and >0.7 for CD204. For each specimen, mean value of the number of cells per mm^2^ in the analysed ROIs was used for further analyses.

### RNAseq

Total RNA was extracted (RNeasy with QIAshredders; Qiagen). RNA quality was assessed by analysis of rRNA band integrity (Agilent RNA 6000 Nano kit; Agilent Technologies, CA, USA). Ahead of cDNA library construction, poly (A) mRNA was enriched by 1 μg of total RNA and magnetic beads with Oligo (dT). Then, the purified mRNAs were disrupted into short fragments, and the double-stranded cDNAs were immediately synthesised. The cDNAs were subjected to end-repair, poly (A) addition, and connected with sequencing adapters (TruSeq RNA sample prep Kit; Illumina, CA, USA). The suitable fragments were automatically purified (BluePippin 2% agarose gel cassette; Sage Science, MA, USA) and were selected as templates for PCR amplification. The final library sizes and qualities were electrophoretically evaluated (High Sensitivity DNA kit; Agilent Technologies, CA, USA), and the fragment was found to be between 350–450 bp. Subsequently, the library was sequenced (HiSeq2500 sequencer; Illumina, CA, USA).

Gene expression level was measured with Cufflinks v2.1.1 using the gene annotation database of Ensembl release 77. Non-coding gene region was removed with the—mask option. To improve the accuracy of measurement, multi-read correction and fragment bias correction options were applied. Abundance estimation ‘-max-bundle-frags’ (maximum number of fragments a locus may have before being skipped) was set to 10,000,000 to estimate the highly-expressed genes. All other options were set to default values.

### Statistical analysis

Continuous variables and proportions were compared using independent T, Mann–Whitney *U*, Chi-square, or Fisher’s exact test, as appropriate. Mean levels of the markers among the three specimen groups were compared using analysis of variance (ANOVA). PFS was defined as the time from the initiation of treatment until the date of documented disease progression or death from any cause, whichever occurred first. For the correlative analysis between TIME and PFS, each marker was stratified by the median value and survival curves were compared using the log-rank test. Two-tailed *P*-values < 0.05 were considered statistically significant. All statistical analyses were performed using SPSS 22.0 software (SPSS Inc., Chicago, IL, USA).

## Results

### Patients

Baseline characteristics of patients are summarised in Table 1. For patients included in this analysis, median age was 55 years (range, 31–76 years), and 61% were male. Small bowel was the most common primary tumour site (*n* = 42, 63%) followed by stomach (*n* = 24, 36%). In 63 patients whose primary genotype was evaluated, *KIT* exon 11 mutation was most common (*n* = 46, 73%) followed by *KIT* exon 9 mutation (*n* = 11, 18%) and *PDGFRA* exon 18 mutation (*n* = 2, 3%). There was no statistically significant difference in sex (*p* = 0.49), age (*p* = 0.29), and primary genotype (*p* = 0.08). All patients included in this analysis received IM. Median PFS with IM was 41.3 months (95% CI, 30.6–52.0) in the TKI-naive group, 34.7 months (95% CI, 18.4–51.0) in the IM-PD group, and 46.7 months (95% CI, 29.7–63.8) in the IM-PD/SU-treated group.

**Table 1:** Baseline patient characteristics

Variables	TKI-naive group (*n* = 20)	IM-PD group (*n* = 30)	IM-PD/SU-treated group (*n* = 31)
Age, years (range)	54 (32–74)	60 (32–76)	50 (31–70)
*Sex*			
Male	15 (75.0%)	18 (60.0%)	19 (61.3%)
Female	5 (25.0%)	12 (40.0%)	12 (38.7%)
*Primary tumour site*			
Stomach	9 (45.0%)	14 (46.7%)	6 (19.4%)
Small bowel	11 (55.0%)	16 (53.3%)	24 (77.4%)
Others	0	0	1 (3.2%)
*Primary mutations*			
*KIT* exon 11	13 (65.0%)	20 (66.7%)	23 (76.7%)
*KIT* exon 9	4 (20.0%)	4 (13.3%)	4 (13.3%)
*PDGFRA* exon 18	0	2 (6.7%)	0
Wild type *KIT/PDGFRA*	2 (10.0%)	2 (6.7%)	0
Not available	1 (5.0%)	2 (6.7%)	3 (10.0%)
*Secondary mutations*		*N* = 17	*N* = 22
*KIT* exon 13		3 (17.6%)	3 (13.6%)
*KIT* exon 14		0	1 (4.5%)
*KIT* exon 17		6 (35.3%)	9 (40.9%)
*KIT* exon 18		0	1 (4.5%)

*TKI* tyrosine kinase inhibitor, IM-PD imatinib-progressed and no exposure to sunitinib or regorafenib, *IM-PD/SU-treated*,  imatinib-progressed and sunitinib and/or regorafenib-treated

### TIME of GISTs in different clinical settings using multiplexed immunofluorescence staining

The number of cells indicating TIME of GISTs per mm^2^ was listed and compared according to the different clinical settings in Table 2. Between the groups (TKI-naive vs IM-PD vs IM-PD/SU-treated), there were statistically significant differences in the number of CD204^+^ CD68^+^ cells (median number of cells per mm^2^ [interquartile range]; 9.1 [0.3–47.3] vs 7.2 [1.8–27.7] vs 23.2 [6.8–128.3] *p* = 0.001); PD-1^+^ CD3^+^ T cells (1.8 [0.3–16.2] vs 0.6 [0.0–6.4] vs 10.4 [3.7–52.2] *p* = 0.02); PD-L1^+^ CD3^+^ T cells (0 [0.0–1.4] vs 0.0 [0.0–0.1] vs 0.9 [0.0–49.3] *p* = 0.02); Ki-67^+^ CD3^+^ T cells (0.3 [0.0–2.3] vs 0.3 [0.1–1.8] vs 7.2 [0.6–17.0] *p* = 0.02); TIM-3^+^ CD3^+^ T cells (0.0 [0.0–1.5] vs 0.0 [0.0–0.4] vs 1.2 [0–6.2] *p* = 0.008); and Ki-67^+^ DOG-1^+^ cells (9.1 [0.2–47.3] vs 104.6 [14.0–207.2] vs 203.6 [44.9–342.0] *p* = 0.02).

**Table 2:** Tumour immune microenvironment according to the clinical setting

Variables	TKI-naive group (*n* = 20), median (IQR 25%–75%)	IM-PD group (*n* = 30), median (IQR 25%–75%)	IM-PD/SU-treated group (*n* = 31), median (IQR 25%–75%)	*P* value
CD3^+^ T cell	112.1 (30.5–575.5)	63.5 (14.1–342.3)	393.0 (88.9–690.0)	0.19
CD8^+^ CD3^+^ T cell	4.9 (1.0–46.1)	4.9 (0.6–26.8)	27.1 (8.0–65.7)	0.16
FoxP3^+^ CD3^+^ cell	3.9 (0.1–24.1)	3.6 (0.6–8.2)	28.0 (6.0–71.1)	0.05
CD68^+^ CD204^+^ cell	9.1 (0.3–47.3)	7.2 (1.8–27.7)	23.2 (6.8–128.3)	**0.001**
CD68^+^ CD204^-^ cell	6.3 (2.6–34.1)	8.5 (4.4–18.8)	15.0 (5.9–56.4)	0.32
PD-1^+^ CD3^+^ T cell	1.8 (0.3–16.2)	0.6 (0.0–6.4)	10.4 (3.7–52.2)	**0.02**
PD-L1^+^ CD3^+^ T cell	0 (0.0–1.4)	0.0 (0.0–0.1)	0.9 (0.0–49.3)	**0.02**
Ki-67^+^ CD3^+^ T cell	0.3 (0.0–2.3)	0.3 (0.1–1.8)	7.2 (0.6–17.0)	**0.02**
Ki-67^+^ CD8^+^ T cell	0.1 (0.0–1.1)	0.5 (0.0–5.2)	5.8 (0.9–20.1)	0.16
TIM-3^+^ CD3^+^ T cell	0.0 (0.0–1.5)	0.0 (0.0–0.4)	1.2 (0–6.2)	**0.008**
LAG-3^+^ CD3^+^ T cell	0.1 (0.0–1.1)	0.1 (0.0–0.7)	0.9 (0.3–6.2)	0.09
PD-L1^+^ DOG-1^+^ tumour cell	0.0 (0.0–7.6)	2.0 (0.6–15.5)	6.9 (0.3–551.7)	0.07
Ki-67^+^ DOG-1^+^ tumour cell	9.1 (0.2–47.3)	104.6 (14.0–207.2)	203.6 (44.9–342.0)	**0.02**

Values are expressed as number of cells per mm^2^

*TKI* tyrosine kinase inhibitor, *IM-PD* imatinib-progressed and no exposure to sunitinib or regorafenib, *IM-PD/SU-treated* imatinib-progressed and sunitinib and/or regorafenib-treated

Bold values indicate statistical significance *p* < 0.05

The PD-L1 expression rate (>1%) of DOG-1^+^ tumour cells was significantly higher in the IM-PD/SU-treated group (29.0%, 9/31) compared to TKI-naive (5.0%, 1/20) and IM-PD (6.7%, 2/30), respectively (*p* = 0.02) (Supplementary Table 2). The expression rate of PD-1 (>1%) in CD3^+^ T cells was 60.0% (12/20), 56.7% (17/30), and 87.1% (27/31), respectively, which was statistically significant (*p* = 0.02).

**Fig. 2:**
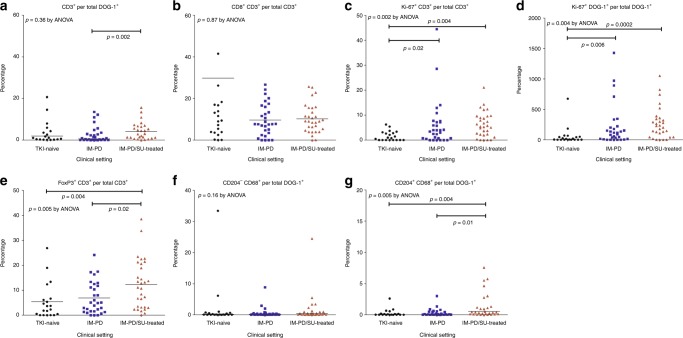
Tumour immune microenvironments according to different clinical settings. TKI, tyrosine kinase inhibitor, IM-PD, imatinib-progressed and no exposure to sunitinib or regorafenib, IM-PD/SU-treated, imatinib-progressed and sunitinib and/or regorafenib-treated **a** CD3^+^ per total DOG-1^+^; **b** CD8^+^ CD3^+^ per total CD3^+^; **c** Ki-67^+^ CD3^+^ per total CD3^+^; **d** Ki-67^+^ DOG-1^+^ per total DOG-1^+^; **e** FoxP3^+^ CD3^+^ per total CD3^+^; **f** CD204^−^ CD68^+^ per total DOG-1^+^; **g** CD204^+^ CD68^+^ per total DOG-1^+^

There was an increase in the rate of CD3^+^ T cells per DOG-1^+^ tumour cells for IM-PD/SU-treated group compared to IM-PD group (*p* = 0.002; Fig. 2a), although CD8^+^ expression of CD3^+^ T cell did not differ between the groups (Fig. 2b). Ki-67 expression of CD3^+^ T cells and DOG-1^+^ tumour cells was significantly higher in IM-PD and IM-PD/SU-treated groups compared to TKI-naive group (CD3^+^ T cells, *p* = 0.02 and *p* = 0.004, respectively (Fig. 2c); and DOG-1^+^ tumour cells, *p* = 0.006 and *p* = 0.0002, respectively (Fig. 2d)); notably, there was no difference between IM-PD and IM-PD/SU-treated groups. FoxP3 expression of CD3^+^ T cells was significantly higher in the IM-PD/SU-treated group compared to TKI-naive group (*p* = 0.004) and IM-PD group (*p* = 0.02: Fig. 2e). Although the rate of CD204^-^ CD68^+^ monocyte cells per DOG-1^+^ tumour cell did not differ by clinical setting (*p* = 0.16; Fig. 2f), the rate of CD204^+^ CD68^+^ monocytes per DOG-1^+^ cell was significantly higher in IM-PD/SU-treated group compared to TKI-naive (*p* = 0.004) and IM-PD groups (*p* = 0.01; Fig. 2g).

**Fig. 3:**
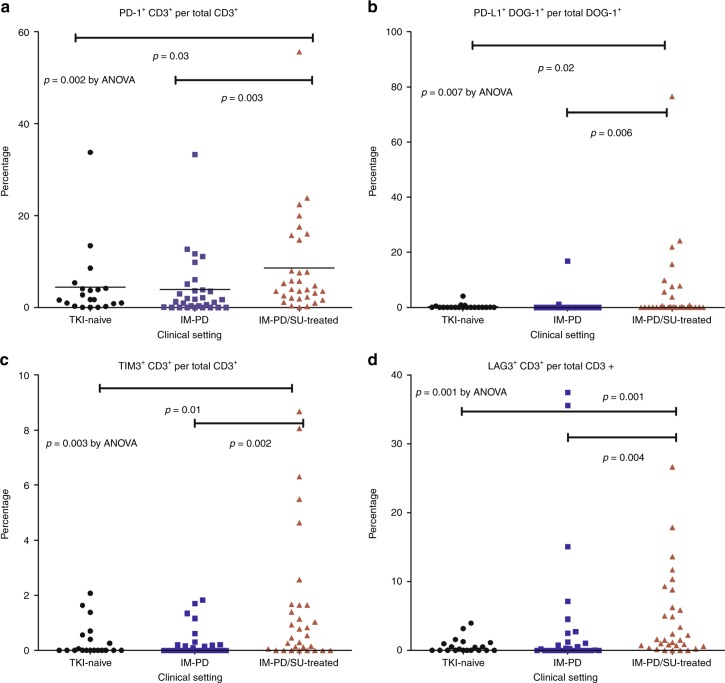
Immune checkpoint molecule expression according to different clinical settings TKI, tyrosine kinase inhibitor, IM-PD group, imatinib-progressed and no exposure to sunitinib or regorafenib, IM-PD/SU-treated, imatinib-progressed and sunitinib and/or regorafenib-treated **a** PD-1^+^ CD3^+^ per total CD3^+^; **b** PD-L1^+^ DOG-1^+^ per total DOG-1^+^; **c** TIM3^+^ CD3^+^ per total CD3^+^; **d** LAG3^+^ CD3^+^ per total CD3^+^

We analysed the status of immune checkpoint molecule expression on CD3^+^ T cells or DOG-1^+^ tumour cells, and these were compared in specimen groups. IM-PD/SU-treated group showed increased expression of immune checkpoint molecules on CD3^+^ T cells or DOG-1^+^ tumour cells compared to TKI-naive or IM-PD groups; PD-1 expression of CD3^+^ T cells (*p* = 0.03 vs TKI-naive and *p* = 0.003 vs IM-PD: Fig. 3a), PD-L1 expression of DOG-1^+^ tumour cells (*p* = 0.02 vs TKI-naive and *p* = 0.006 vs IM-PD: Fig. 3c), TIM-3 expression of CD3^+^ T cells (*p* = 0.01 vs TKI-naive and *p* = 0.002 vs IM-PD: Fig. 3d), LAG3 expression of CD3^+^ T cells (*p* = 0.001 vs TKI-naive and *p* = 0.004 vs IM-PD: Fig. 3e).

### Gene expression analysis using RNAseq in different clinical settings

RNAseq was performed for TKI-naive (*n* = 10), IM-PD (*n* = 14), and IM-PD/SU-treated (*n* = 5) groups. Results of gene expression analysis using RNAseq in different clinical setting are depicted in Supplementary Fig. 1. FoxP3 expression was marginally increased in IM-PD/SU-treated group compared to TKI-naive group (*p* = 0.11). IM-PD/SU-treated GISTs had marginal increase in TIM-3 expression compared to IM-PD GISTs (*p* = 0.06). TIGIT expression was significantly increased in IM-PD/SU-treated group compared to IM-PD group (*p* = 0.01).

### Correlative analysis between TIME and PFS with IM

**Fig. 4:**
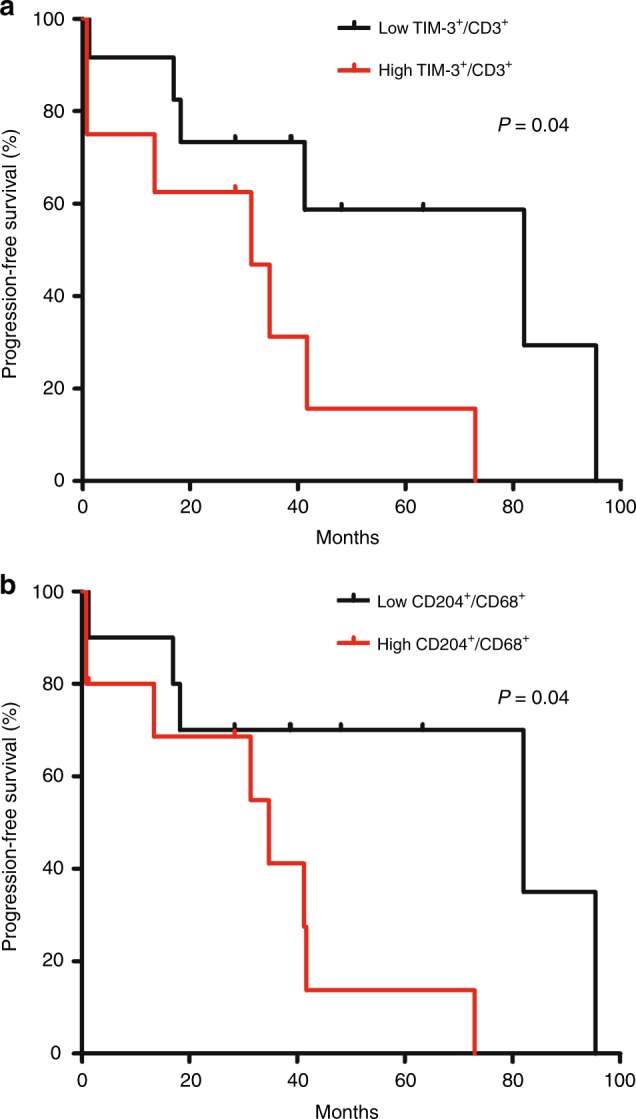
Association between tumour immune microenvironment and progression-free survival with imatinib. **a** TIM-3^+^ CD3^+^; **b** CD204^+^ CD68^+^

In TKI-naive group, correlative analysis for the relationship between TIME and PFS with IM was performed. Each marker was classified as high (≥median) vs low (<median) groups. High TIM-3^+^ CD3^+^ T cells and CD204^+^ CD68^+^ monocytes were significantly associated with poorer PFS with IM (*p* = 0.04 for both; Fig. 4). Otherwise, there was no significant correlation between other types of TIME and PFS with IM (Supplementary Fig. 2).

## Discussion

GIST is one of the representative diseases for the successful introduction of targeted therapy including IM, SU and REG that has facilitated a remarkable improvement in survival. Novel compounds have shown promising data on IM-refractory GISTs in the early phases of clinical trials^9,10^; however, new drug development in advanced GISTs still mainly targets the KIT inhibition despite the emergence of immunotherapy in other types of cancers. Considering that the cancer immune microenvironment may be closely related to effective strategies in immunotherapy, a better understanding of the GIST-specific TIME is essential for better therapeutic development using immunotherapeutic agents.

In this study, we performed multiplexed immunofluorescence staining and RNAseq on human GIST specimens to characterise the immune landscape of GISTs in different clinical settings. Current analysis reaffirms that GISTs harbour the tumour infiltration of immune cells, including various types of T cells and macrophages. Numbers of pan-T cell infiltrations (CD3^+^ cells) and macrophages (CD68^+^ cells) of TKI-naive GISTs were comparable to those in a previous report.^15^ Although another previous study presented that tumour PD-L1 expression (29%) and lymphocyte PD-L1 expression (50%) were relatively common in GISTs,^12^ our study showed that cytotoxic T cells (CD8^+^ CD3^+^), T cells with exhausted phenotype (regulatory T cells [FoxP3^+^ CD3^+^], PD-L1^+^ T cells, and TIM-3^+^ T cells) were scarce in TKI-naive GISTs. Although discrepancies between the current and previous studies may be due to variability in the PD-L1 assays and assessment methodologies,^12^ this may be a result of the different patient characteristics among the studies. In our study, all patients in the TKI-naive group progressed later, which required systemic therapy; however, previous studies included heterogeneous patient populations that included early-stage disease. There is a report that immune cells are highly infiltrated in localised GISTs with good prognosis compared to those with poor prognosis.^13^ Thus, our TKI-naive cohort that progressed and subsequently required systemic therapy may have less immune cell infiltrates or immune-exhausted phenotypes compared to previous studies. Our TKI-naive cohort may be more clinically relevant than unselected patient populations (including those with very early disease or low risk of recurrence) for analysis of the TIME to find the potential target or implication of immunotherapy against treatment-naive metastatic or unresectable GISTs.

Our findings suggest that the TIME of GISTs may be affected by systemic anti-cancer treatment. Exhausted T cell phenotypes were prominent in the group treated with anti-angiogenic agents (i.e., IM-PD/SU-treated group) compared to TKI-naive and IM-PD groups, while there was no significance differences of TIME profiles between TKI-naive and IM-PD groups. Expression of FoxP3, PD-1, TIM-3, and LAG-3 in CD3^+^ T cells was significantly higher in multiplexed immunofluorescence staining, and RNAseq analysis also supported these results. PD-L1 expression of tumour cells was also higher in the IM-PD/SU-treated group compared to TKI-naive and IM-PD groups. These findings may suggest that GISTs progressed on IM and treated with SU or REG are the better potential candidates for future clinical trials of ICIs against advanced GISTs, as high PD-L1 expression of tumour or immune cells was correlated with better efficacy with anti-PD-1 or anti-PD-L1 inhibitors in other cancer types.^16,17^ Based on the design of the current study, our results do not preclude the possibility that these changes are a result of natural course of disease progression. Ki-67 expression, an indicator of proliferation of immune cells and tumour cells, did not differ between the IM-PD/SU-treated and IM-PD groups; however, there was a significant increase in the expression of immune checkpoint molecules on CD3^+^ T cells and PD-L1 expression of DOG-1^+^ tumour cells in the IM-PD/SU-treated group compared to the IM-PD group. This reinforces the possibility that treatment with SU or REG, anti-angiogenic TKIs, modifies the TIME of GISTs after progression on IM. Recent studies have shown that anti-angiogenic agents promote T cell infiltration into a tumour.^18^ In addition, anti-angiogenic agents may have a role in enhancing the efficacy of immunotherapy via reversing the immunosuppressive tumour microenvironment.^19–21^

Our analysis also showed that M2-polarised macrophage (CD204^+^ CD68^+^ cells) was increased in IM-PD/SU-treated GISTs compared to TKI-naive and IM-PD GISTs, while there was no difference in M1-polarised macrophage (CD204^−^ CD68^+^ cells) among the groups. This analysis is consistent with the results of previous studies that showed that M2-polarised macrophage was more dominant in TKI-treated GISTs compared to TKI-naive GISTs.^15^ Macrophage with M2 phenotype has been known to have an anti-inflammatory effect and consists of most tumour-associated macrophages (TAM).^22^ M2-polarised macrophages also exhibit functions that may help tumour progression, and they show a negative correlation with survival outcomes in patients with advanced solid tumours.^23,24^ A previous study showed that M2-polarised macrophage was more commonly found in metastatic GIST compared to primary lesions. As a result, increased M2-polarised macrophage in IM-PD/SU-treated GISTs may be related with the tumour progression itself.^15^ However, considering the interaction between macrophage polarisation and angiogenesis, the potential impact of SU or REG on this phenomenon cannot be excluded.^25^

Immunotherapy, particularly with ICIs, such as PD-1 or PD-L1 inhibitors or CTLA-4 inhibitors, has changed the entire paradigm for the therapeutic landscape of multiple cancer types. However, inhibition of c-KIT via IM, SU, and REG remains a major therapeutic strategy against advanced GISTs. Although several small trials using ICIs, such as nivolumab, nivolumab plus ipilimumab, and ipilimumab plus dasatinib have been conducted for patients with advanced GISTs, the outcomes of these regimens are unlikely to be promising in unselected patient populations.^26^ Based on our current findings, ICI monotherapy or a combination therapy of multiple ICIs in patients with GISTs who have previously used anti-angiogenic agents may be a valuable strategy to be investigated, as immune checkpoint molecules are overexpressed on T cells and tumour cells in this patient population. Considering the immune-modulating effect of anti-angiogenic agents in GISTs demonstrated in our study, a combination therapy of ICIs and anti-angiogenic agents may be a potential approach to enhance the efficacy of ICIs in advanced GISTs. Indeed, this strategy has shown successful preliminary outcomes in advanced hepatocellular carcinoma.^27^ In addition to T cell-targeted immunotherapy, targeting tumour-associated macrophages via CCR2, CSF1R, or CD40 may be effective in patients with GISTs who have previously used anti-angiogenic agents, as M2 macrophages were increased in this patient population. These agents showed promising preliminary outcomes in other cancer types.^28–30^

In TKI-naive group, greater tumour infiltration of TIM-3^+^ CD3^+^ T cells and M2-polarised macrophages (CD204^+^ CD68^+^ cells) were significantly associated with poorer PFS with IM. This is in line with the results of prior studies for other cancer types^23,24^ and is interesting finding which may indicate that the pattern of TIME may be predictive for the outcomes with IM. Although this should be cautiously interpreted and further validation in a larger patient population is required because of the small number of patients included in the correlative analysis, this may imply the potential usefulness of targeting TIM-3 or M2-polarised macrophages as a combination therapy with the backbone of IM.

A recent immune profiling study based on human GIST samples revealed that *PDGFRA*-mutant GISTs contained more immune cells than *KIT*-mutant GISTs, which suggested that patients with *PDGFRA*-mutant GISTs could have the potential to respond to ICIs.^31^ Because only two cases with *PDGFRA* mutations were included, we could not validate these findings in the current analysis.

One of the limitations in our study is that RNAseq was performed in the very small number of patients; therefore, data of RNAseq were underpowered to find significant findings by itself and only used to support the findings from multiplexed immunofluorescence staining. Because RNAseq has more stringent quality checkpoints to ensure the reliable data interpretation compared to immunohistochemistry, only limited number of patients included in this study could perform RNAseq.

In conclusion, the immunosuppressive phenotype was predominant in tumours treated with anti-angiogenic agents compared to tumours with TKI-naive and IM treatment. This suggests that this patient population may be a good candidate for future immunotherapy clinical trials targeting T cells or macrophages. Further investigations are needed to determine the optimal immunotherapy strategy in specific GIST subpopulations.

## Supplementary information

Supplementary Figure and Table

## Data Availability

The data that support the findings of this study are available from the corresponding author upon reasonable request.

## References

[1] Joensuu H, Hohenberger P, Corless CL (2013). Gastrointestinal stromal tumour. Lancet.

[2] Demetri GD, Mehren von M, Blanke CD, Van den Abbeele AD, Eisenberg B, Roberts PJ (2002). Efficacy and safety of imatinib mesylate in advanced gastrointestinal stromal tumors. N. Engl. J. Med..

[3] Blanke CD, Demetri GD, Mehren von M, Heinrich MC, Eisenberg B, Fletcher JA (2008). Long-Term Results From a Randomized Phase II Trial of Standard- Versus Higher-Dose Imatinib Mesylate for Patients With Unresectable or Metastatic Gastrointestinal Stromal Tumors Expressing KIT. J. Clin. Oncol..

[4] Demetri GD, van Oosterom AT, Garrett CR, Blackstein ME, Shah MH, Verweij J (2006). Efficacy and safety of sunitinib in patients with advanced gastrointestinal stromal tumour after failure of imatinib: a randomised controlled trial. Lancet.

[5] Demetri GD, Reichardt P, Kang Y-K, Blay J-Y, Rutkowski P, Gelderblom H (2013). Efficacy and safety of regorafenib for advanced gastrointestinal stromal tumours after failure of imatinib and sunitinib (GRID): an international, multicentre, randomised, placebo-controlled, phase 3 trial. Lancet.

[6] Kang Y-K, Ryu M-H, Yoo C, Ryoo B-Y, Kim HJ, Lee JJ (2013). Resumption of imatinib to control metastatic or unresectable gastrointestinal stromal tumours after failure of imatinib and sunitinib (RIGHT): a randomised,placebo-controlled, phase 3 trial. Lancet Oncol.

[7] Corless CL, Barnett CM, Heinrich MC (2011). Gastrointestinal stromal tumours:origin and molecular oncology. Nat. Rev. Cancer.

[8] Heinrich MC (2003). Kinase mutations and imatinib response in patients with metastatic gastrointestinal stromal tumor. J. Clin. Oncol..

[9] Robert C, Thomas L, Bondarenko I, O’Day S, Weber J, Garbe C (2011). Ipilimumab plus dacarbazine for previously untreated metastatic melanoma. N. Engl. J. Med..

[10] Wolchok JD, Kluger H, Callahan MK, Postow MA, Rizvi NA, Lesokhin AM (2013). Nivolumab plus Ipilimumab in Advanced Melanoma. N. Engl. J. Med..

[11] Robert C, Schachter J, Long GV, Arance A, Grob JJ, Mortier L (2015). Pembrolizumab versus Ipilimumab in Advanced Melanoma. N. Engl. J. Med..

[12] D’Angelo SP, Shoushtari AN, Agaram NP, Kuk D, Qin L-X, Carvajal RD (2015). Prevalence of tumor-infiltrating lymphocytes and PD-L1 expression in the soft tissue sarcoma microenvironment. Human Pathol.

[13] Rusakiewicz S, Semeraro M, Sarabi M, Desbois M, Locher C, Mendez R (2013). Immune infiltrates are prognostic factors in localized gastrointestinal stromal tumors. Cancer Res.

[14] Binnewies M, Roberts EW, Kersten K, Chan V, Fearon DF, Merad M (2018). Understanding the tumor immune microenvironment (TIME) for effective therapy. Nat. Med..

[15] van Dongen M, Savage NDL, Jordanova ES, Briaire-de Bruijn IH, Walburg KV, Ottenhoff THM (2010). Anti-inflammatory M2 type macrophages characterize metastasized and tyrosine kinase inhibitor-treated gastrointestinal stromal tumors. Int. J. Cancer.

[16] McDermott DF, Sosman JA, Sznol M, Massard C, Gordon MS, Hamid O (2016). Atezolizumab, an anti–programmed death-ligand 1 antibody, in metastatic renal cell carcinoma: long-term safety, clinical activity, and immune correlates from a phase ia study. J. Clin. Oncol..

[17] Daud AI, Wolchok JD, Robert C, Hwu W-J, Weber JS, Ribas A (2016). Programmed death-ligand 1 expression and response to the anti–programmed death 1 antibody Pembrolizumab in melanoma. J. Clin. Oncol..

[18] Bendell JC, Funke R, Sznol M, Korski K, Jones S, Hernandez G (2016). Atezolizumab in combination with bevacizumab enhances antigen-specific T-cell migration in metastatic renal cell carcinoma. Nat. Comms..

[19] Motz GT, Santoro SP, Wang L-P, Garrabrant T, Lastra RR, Hagemann IS (2014). Tumor endothelium FasL establishes a selective immune barrier promoting tolerance in tumors. Nat. Med..

[20] Hegde PS, Wallin JJ, Mancao C (2018). Predictive markers of anti-VEGF and emerging role of angiogenesis inhibitors as immunotherapeutics. Semin. Cancer Biol..

[21] Goel S, Duda DG, Xu L, Munn LL, Boucher Y, Fukumura D (2011). Normalization of the vasculature for treatment of cancer and other diseases. Physiol. Rev..

[22] Brown JM, Recht L, Strober S (2017). The promise of targeting macrophages in cancer therapy. Clin. Cancer Res..

[23] Steidl C, Lee T, Shah SP, Farinha P, Han G, Nayar T (2010). Tumor-associated macrophages and survival in classic Hodgkin’s lymphoma. N. Engl. J. Med..

[24] Ostuni R, Kratochvill F, Murray PJ, Natoli G (2015). Macrophages and cancer: from mechanisms to therapeutic implications. Trends Immunol..

[25] De Palma M, Biziato D, Petrova TV (2017). Microenvironmental regulation of tumour angiogenesis. Nat. Rev. Cancer.

[26] D’Angelo SP, Shoushtari AN, Keohan ML, Dickson MA, Gounder MM, Chi P (2017). Combined KIT and CTLA-4 Blockade in patients with refractory GIST and other advanced sarcomas: a phase ib study of dasatinib plus ipilimumab. Clin. Cancer Res..

[27] Ikeda M, Sung MW, Kudo M, Kobayashi M, Baron AD, Finn RS (2018). A phase 1b trial of lenvatinib (LEN) plus pembrolizumab (PEM) in patients (pts) with unresectable hepatocellular carcinoma (uHCC). J. Clin. Oncol..

[28] Beatty GL, Chiorean EG, Fishman MP, Saboury B, Teitelbaum UR, Sun W (2011). CD40 Agonists alter tumor stroma and show efficacy against pancreatic carcinoma in mice and humans. Science.

[29] Zhu Y, Knolhoff BL, Meyer MA, Nywening TM, West BL, Luo J (2014). CSF1/CSF1R Blockade reprograms tumor-infiltrating macrophages and improves response to t-cell checkpoint immunotherapy in pancreatic cancer models. Cancer Res..

[30] Nywening TMN, Wang-Gillam AW-G, Sanford DES, Belt BAB, Panni RZP, Cusworth BMC (2016). Targeting tumour-associated macrophages with CCR2 inhibition in combination with FOLFIRINOX in patients with borderline resectable and locally advanced pancreatic cancer: a single-centre, open-label, dose-finding, non-randomised, phase 1b trial. Lancet Oncol.

[31] Vitiello GA, Bowler TG, Liu M, Medina BD, Zhang JQ, Param NJ (2019). Differential immune profiles distinguish the mutational subtypes of gastrointestinal stromal tumor. J. Clin. Invest..

