# Supplementary material for: Association between the exposure to anti-angiogenic agents and tumour immune microenvironment in advanced gastrointestinal stromal tumours
Source: Br J Cancer. 2019 Oct 14;121(10):819–26. doi: 10.1038/s41416-019-0596-1 (PMC6888806; doi:10.1038/s41416-019-0596-1)

**Supplementary Table 1. Panel information**

|  | **Antibody** | **Clone** | **Ab Titration** | **TSA** | **Titration** |
| --- | --- | --- | --- | --- | --- |
| Panel 1 | DOG1 | SP31, Cell Marque | 1:300 | Opal520 | 1:150 |
|  | CD3 | 2GV6, Ventana | 1:5 | Opal540 | 1:150 |
|  | CD8 | 4B11, AbD Serotec | 1:300 | Opal570 | 1:300 |
|  | Foxp3 | 236A/E7, Abcam | 1:100 | Opal620 | 1:150 |
|  | PD-L1 | E1L3N^®,^ CST | 1:300 | Opal650 | 1:300 |
|  | PD-1 | EPR4877, AbCam | 1:300 | Opal690 | 1:150 |
| Panel 2 | DOG1 | SP31, Cell Marque | 1:300 | Opal520 | 1:150 |
|  | CD3 | 2GV6, Ventana | 1:5 | Opal540 | 1:150 |
|  | CD68 | PG-M1, Dako | 1:2000 | Opal570 | 1:300 |
|  | TIM-3 | D5D5R^TM^, CST | 1:200 | Opal650 | 1:150 |
|  | CD204 | SRA-E5, TransGenic | 1:400 | Opal690 | 1:300 |
| Panel 3 | DOG1 | SP31, Cell Marque | 1:300 | Opal520 | 1:150 |
|  | CD3 | 2GV6, Ventana | 1:5 | Opal540 | 1:150 |
|  | LAG-3 | 17B4, LSBio | 1:100 | Opal620 | 1:150 |
|  | Ki67 | MIB-1, Dako | 1:200 | Opal650 | 1:150 |
|  | CD8 | 4B11, AbD Serotec | 1:300 | Opal690 | 1:300 |

Ab titration = antibody titration, TSA = tyramide signal amplification

**Supplementary Table 2. PD-L1 and PD-1 expression rates of tumor and immune cells**

| Variables | TKI-naïve group (n = 20) | IM-PD group (n = 30) | IM-PD/SU-treated group (n = 31) | P value |
| --- | --- | --- | --- | --- |
| Proportion of > 1% PD-L1^+^ DOG-1^+^ tumor cells /DOG-1^+^ tumor cells | 1 (5.0%) | 2 (6.7%) | 9 (29.0%) | 0.02 |
| Proportion of > 1% PD-1^+^ CD3^+^ cells /CD3^+^ cells | 12 (60.0) | 17 (56.7%) | 27 (87.1) | 0.02 |

**Supplementary Figure 1. Gene expression analysis using RNAseq according to different clinical settings**

TKI=tyrosine kinase inhibitor, IM-PD group= imatinib-progressed and no exposure to sunitinib or regorafenib, IM-PD/SU-treated = imatinib-progressed and sunitinib and/or regorafenib-treated


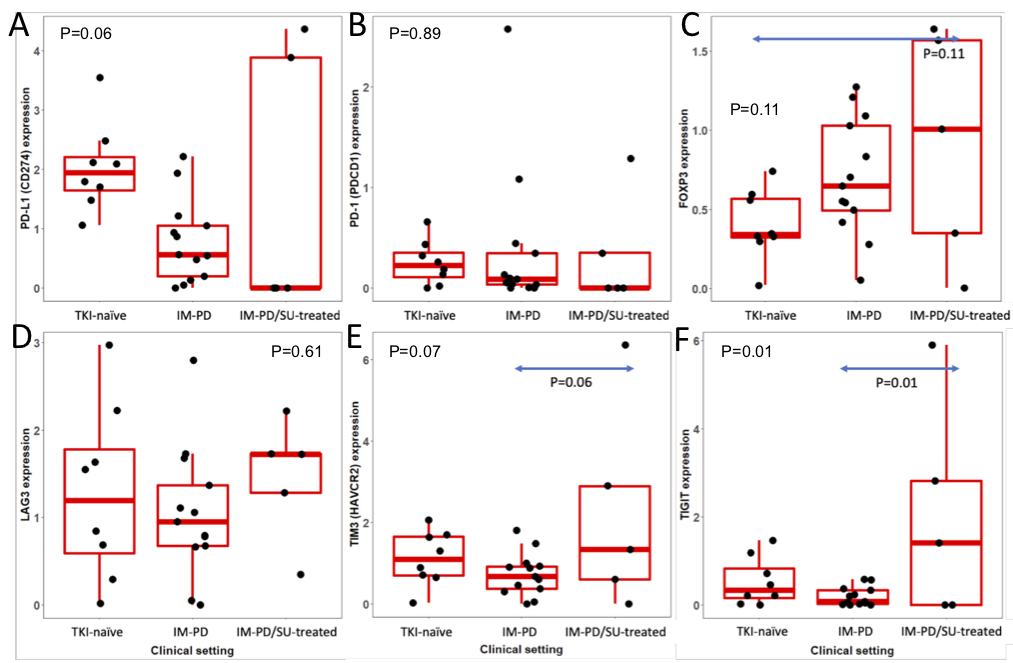


**Supplementary Figure 2. Correlative analysis between tumor immune microenvironment and progression-free survival with imatinib in the TKI-naïve group**

TKI=tyrosine kinase inhibitor


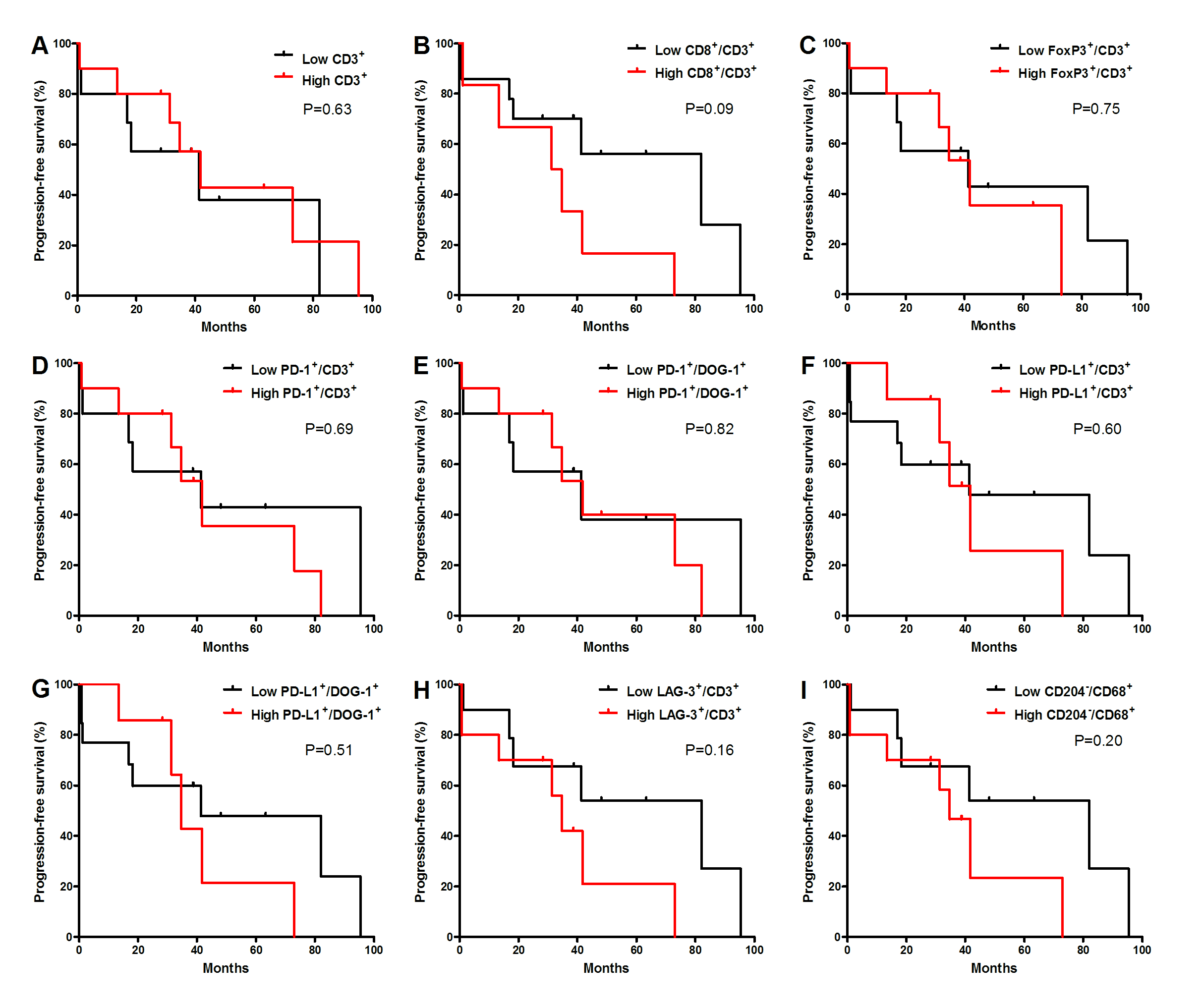

Supplement: Supplementary file 1 — Supplementary Figure and Table [file 41416_2019_596_MOESM1_ESM.docx]
